# Creating an Efficient Point-of-Care Ultrasound Workflow

**DOI:** 10.24908/pocus.v5i2.14429

**Published:** 2020-11-18

**Authors:** Ammar Saati, Arthur Au, Titus Chu, Rebecca L Davis, Rohin Singla, Jason Smith, Jennifer L White, Resa E Lewiss

**Affiliations:** 1 Thomas Jefferson University Philadelphia, PA USA

**Keywords:** POCUS, workflow

## Letter:

A point-of-care ultrasound (POCUS) workflow is composed of multiple processes managed by various stakeholders. There are concurrent front and back end steps including: acquiring, archiving and interpreting images; documenting the POCUS study and ultimately coding and billing for the study 1. An efficient POCUS workflow is important for both patient care and billing 2, 3, 4. Unfortunately, there is no single standard across POCUS programs 5. Based on our institutional experience, we suggest that programs engage key groups described in this perspective piece. 

The Clinical Operations team includes the clinicians, who serve in the role of the Vice Chair of Operations, the Medical Director for the hospital site, the Nurse Manager, and others employed to maintain and improve the day-to-day functioning of the department. This is the most important group with whom to partner. For the sake of illustration, consider an electrocardiogram (ECG) workflow. What happens when an ECG machine breaks? The operations team, who is responsible for the machinery, works closely with Biomedical engineering (Biomed) and information systems and technology (IS&T) to get the ECG machine functional, online, and interfacing. Ideally, there is a clearly defined process for escalating real time technical issues to the operations team, that does not require the POCUS team to be directly involved. 

The Biomed team is primarily responsible for ultrasound machine hardware. Although most readily available during business hours, they are available 24 hours a day and 7 days a week. These team members have the technical expertise to handle ultrasound equipment issues, including cords and transducers. They also interface directly with the ultrasound companies. They can, for example, navigate loaner machines and transducer exchanges. Biomed can also assist with routine maintenance of machinery, such as cleaning fans, or adjusting clocks during daylight savings. 

The IS&T team maintains the wifi connectivity of the ultrasound machines as well as the transmission and storage of POCUS examinations. Involving this team is crucial for any connectivity problems which arise: Electronic Health Record (EHR) to machine, machine to wifi transmission, and machine to the picture archiving and communication system PACS 6. The IS&T team monitors the work process, and optimizes efficiency 7. 

The POCUS faculty are the primary point of communication with the EHR and billing teams. The EHR team is rarely an active participant in the workflow once launched. When first creating the workflow, this team is required to ensure procedure note creation specific to institutional POCUS criteria and coding/billing guidelines. This team is particularly important for automating certain tasks, such as sending reminders to clinicians, who perform an examination but forget to document their POCUS examination. 

On the back end of building the workflow is the coding and billing team. Once coding and billing are established for the POCUS program, this team can interface with the department administration and POCUS faculty for periodic e.g. monthly reviews to maximize compensation for the patient care being performed 4.

Often, not enough expectation and responsibility is placed on the clinician performing the POCUS examination. The culture and messaging is best led by the department chairperson and leadership alongside the POCUS faculty leadership. Using the analogy of the ECG again, the clinician knows his/her responsibility in ordering, reading, documenting, and integrating the ECG interpretation. Similarly, the clinician's adherence to a POCUS workflow and standardized protocol is a key element of continued success. Creating fail-safes in the workflow, including EHR alerts and automated messaging to encourage standardization in behaviors can improve clinician adherence with departmental expectations. Clinicians also should be educated and reminded of the POCUS workflow, so that if they encounter breaks in the system, they know who to contact and how to get problems addressed realtime. Regular clinician specific feedback and metrics sharing are important.

A POCUS workflow can be an efficient process, and in our experience this requires the communication, commitment, and involvement of a multidisciplinary team. We realize that what we propose requires a culture change for many institutions with historically established silos of responsibility. However, we believe that keeping the focus of the workflow on delivering timely, safe, and cost-efficient patient care is the foundation on which to build. Team building, communication, and the delegation of responsibilities, based upon subject matter expertise, can facilitate an efficient POCUS workflow. 

## Conflicts of Interest

None declared.
